# Zeolite Additives for Flexible Packaging Polymers: Current Status Review and Future Perspectives

**DOI:** 10.3390/polym16233399

**Published:** 2024-12-02

**Authors:** Mattia Fornaro, Barbara Liguori, Veronica Ambrogi, Domenico Caputo

**Affiliations:** 1 ACLabs—Applied Chemistry Labs, Department of Chemical, Materials and Industrial Production Engineering, University of Naples Federico II, P.le Tecchio 80, 80125 Napoli, Italy; mattia.fornaro@unina.it (M.F.);; 2 National Interuniversity Consortium of Materials Science and Technology (INSTM), Via G. Giusti 9, 50121 Firenze, Italy; 3 PolyFun, Department of Chemical, Materials and Industrial Production Engineering, University of Naples Federico II, P.le Tecchio 80, 80125 Napoli, Italy

**Keywords:** packaging, additives, zeolites, active, polyolefin, biodegradable

## Abstract

Zeolites are interesting inorganic additives that could be employed for plastic packaging applications. Polyethylene (PE) and polypropylene (PP) are intensively used for packaging as they provide great performance at low cost, even though they have poor environmental sustainability and may be more valorized. Biodegradable polymers may therefore represent a more eco-friendly alternative, but still, they have limited applications due to their generally inferior properties. Therefore, this review focuses on the use of zeolites as additives for flexible packaging applications to mainly improve the mechanical and barrier properties of PE, PP, and some biodegradable polymers, possibly with antimicrobial and scavenging activities, by exploiting zeolites’ cation exchange ability and adsorption properties. Film preparation and characterization have been investigated. The obtained enhancements regard generally higher gas barriers, elastic moduli, and strengths, along with thermal stability. Elongation at break decreased for all PE composites and tended to increase for other matrices. The use of zeolites as additives for polymer films is promising (mainly for biodegradable polymers); still, it requires overcoming some limiting drawbacks associated with the additive concentration and dispersion mainly due to matrix–additive incompatibility.

## 1. Introduction

Packaging refers to any material used to contain and protect goods, to allow their handling and delivery from the producer to the consumer [1]. Packaging can be further classified into rigid—thick, with high mechanical protection and strong barrier properties—and flexible—light, thin, and generally cheaper [2]. Additionally, there is a strong interest in active food packaging, which involves interactions between the package and the internal gas atmosphere or directly with food, in order to extend the shelf life of goods and inhibit the growth of spoilage microorganisms [3,4].

Plastics are the most used materials for packaging, mainly for food and beverage applications. Globally, one-third of the total packaging market is represented by flexible plastics and one-fifth by rigid plastics, followed by paper/paperboard, metal, and glass. Plastics can be used as layers (cast or co-extruded) in flexible/rigid polylaminate packages and as flexible self-standing packaging films [2,5,6,7,8]. For instance, interesting sustainable and recyclable packaging materials are paper and paperboard; however, these may provide inadequate barrier properties, heat sealability, and strength, unless they are treated with additives or laminated, mainly with plastics [9,10].

Therefore, plastics (even as thin layers) are usually unavoidable in packaging applications. Enhancing their technological and barrier properties and thus valorizing their life cycle is crucial.

### 1.1. Conventional Polyolefins: PE and PP

The largest market shares in packaging materials belong to low-cost, commodity thermoplastic polymers, especially polyolefins, such as polyethylene (PE) and polypropylene (PP). Polyolefins belong to the class of addition polymers and are particularly durable, with strong chemical and biological inertness, because of their high molecular weight, hydrophobicity, and absence of functional reactive groups. Furthermore, their properties can be easily adjusted to achieve the desired technological properties such as strength, permeability, porosity, opacity, and color [11,12].

**Figure 1:**
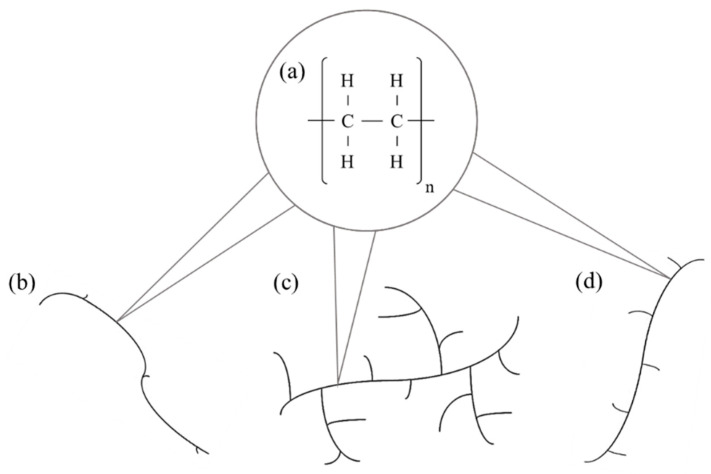
Scheme of (**a**) the repeating unit of PE; schemes of the main molecular structures of PE differing in linearity and branching: (**b**) HDPE, (**c**) LDPE, and (**d**) LLDPE.

As of 2021, PE represents the most produced polymer worldwide, mainly used for several packaging applications [8]. This class of polyolefins is very suitable for food packaging films: it has high processability and provides great water vapor barriers (but low O_2_ barrier) [13]. PE needs to be classified into two main different polymeric structures that affect final film performances: low-density polyethylene (LDPE), with many and long side-chain branches, softer and more flexible, with good sealability, and high-density polyethylene (HDPE), linear, with few and short side-chain branches, with higher stiffness and resistance [13,14,15]. Then, under the name, linear low-density polyethylene (LLDPE), a family of ethylene-based copolymers, is represented, consisting of long and linear PE chains with many short side-chain branches of other alkene co-monomers: they are excellent sealants with high strength and toughness [13,14,15,16]. The structures of LDPE, HDPE, and LLDPE are schematized in Figure 1.

PP ranks as the second most widely produced polymer, primarily demanded for its applications in packaging [8]. It is semicrystalline and relatively stiff with high chemical inertness and melting point, has good barrier properties (intermediate to that of LDPE and HDPE), and has high resistance to impacts [13,17,18,19].

These commodity polymers are conventionally petroleum-based; still, bio-based alternatives exist—with “bio-based” meaning that these polymers can be obtained from either biomass or renewable resources [20]. However, due to their cost, the production of bio-based counterparts is negligible compared to that of conventional polyolefins [21].

Since conventional petroleum-based polyolefins are strongly spread worldwide for packaging, these polymers—usually produced to be disposable—represent a major type of pollution among all types of plastics, with PE wastes that are a primary aware, mainly for the marine environment [22,23]. Still, mono-material PE and PP flexible packages can be easily and successfully mechanically recycled, differently from polylaminate multi-material films [24,25], if end of life is correctly managed and a certain waste homogeneity is achieved [26]. 

### 1.2. Biodegradable Plastics

Some polymers, regardless of their being petroleum- or bio-based, can also be biodegradable, meaning that environmental microorganisms can convert them into natural substances such as CO_2_, H_2_O, and CH_4_, depending on whether the process is aerobic or anaerobic. Factors influencing biodegradation include environmental conditions, the chemistry of the polymer, and its specific application. Standards are internationally used to assess the biodegradability of these polymers: the compliance with certain biodegradation rates under different specific conditions has to be certified using standardized test methods, such as the ones provided by ASTM [27,28,29]. Furthermore, some biodegradable polymers can also be classified as compostable, meaning they can fully degrade into the soil as compost according to standards [30]. Not all biodegradable plastics are compostable, as composting is not natural but a human-driven process under optimized conditions [31,32]. PE and PP, due to their chemistry, are not biodegradable [20]. Nevertheless, it is important to disclose that biodegradable polymers—generally protein-based or carbohydrate-based—have low physical barrier properties when compared to conventional petroleum-based polymers (such as PE and PP), and additionally, their sealability (a key step in packaging) is hard to achieve. However, research moves on functionalizing these materials and valorizing their potential [10]. Chitosan, polylactic acid (PLA), gelatin, thermoplastic starch (TPS), and polybutylene succinate (PBS) are biodegradable polymers, mostly bio-based and compostable, which can be potentially used for eco-friendly packaging applications, both as self-standing films (analogously to PE and PP) and as coatings. 

Starch is a polysaccharide made up of amylose (linear chain of glucose repeating units, 15–20%) and amylopectin (branched molecule made of several thousands of glucose units, 80–85%) [33]. Due to its structure, native starch cannot be processed as a thermoplastic film; however, with plasticizers, a deformable biodegradable and compostable material, called thermoplastic starch (TPS), can be obtained [34,35]. Still, due to high moisture sensitivity and critical aging, TPS alone has limited applications [36].

PLA is a biodegradable and compostable thermoplastic polyester [37]. It is provided with excellent thermoforming ability, and its films have high mechanical strength, great optical properties, and biocompatibility; still, its inherent rigidity and brittleness, along with high water vapor permeability, currently limit neat PLA applications compared to conventional polyolefins [38,39,40,41,42]. Therefore, in order to improve the technological properties required for packaging applications, the employment of (properly chosen) plasticizers is required for PLA to increase flexibility, ductility, and resistance to impacts [43].

Chitosan and chitosan oligomers are polysaccharides extracted from shrimp shells that are receiving much interest thanks to their biocompatibility, antimicrobial activity, and film-forming properties [44,45,46,47]. Chitosan films are transparent, possibly strong and flexible, and have low oxygen permeability. These films, however, have a certain permeability to water vapor, and since chitosan is not thermoplastic, it cannot be heat-sealed. Additionally, chitosan is generally obtained by solvent casting from an acid solution, as this polymer is poorly soluble in neutral media, and this may also limit its applications [48,49,50].

Pectin is another interesting polysaccharide for packaging applications, extracted from fruits and vegetables. According to the esterification degree, pectin can be divided into high methoxy (HM) or low methoxy pectin (LM). Pectin films, easily obtainable by solution casting, are edible [51,52,53]. However, due to its high solubility in water and poor technological properties, pectin has not found wide packaging applications yet, demanding the use of fillers to enhance these film properties [52,54].

Gelatin is a green edible protein-based polymer that is extracted from collagen in animal sources or organs. It can provide good film-forming properties and biodegradability, along with reported antibacterial properties. Properly synthesized gelatin films show a strong gas barrier against O_2_ and CO_2_; however, weak mechanical resistance and very high permeability to water vapor molecules limit the possible application of this polymer [55,56]. Still, if plasticized, gelatin can form enough robust and flexible films [57,58].

To conclude, another interesting polymer for packaging applications is PBS, an aliphatic polyester that has been considered a biodegradable alternative to PE and PP, as they share similar mechanical, thermal, and processing properties. The monomer is conventionally obtained from petrochemicals; still, bio-based PBS can be produced. It provides good thermal properties, melt processability, and chemical resistance. Nonetheless, as a neat polymer, it does exhibit brittleness and low gas barrier properties [33,59,60,61].

Nowadays, the majority of the globally produced biodegradable polymers are used for packaging, mostly PLA. It is worth saying that less than 0.3% of globally produced plastic products are made of biodegradable polymers; still, sensible growth is expected in the next years [21,62].

### 1.3. Additives in Packaging

Additives are used to enhance neat films’ performances and enrich them with new interesting technological properties. Increasing the performance and durability of these polymers may also reduce the overall request for virgin polymers by valorizing them. Commonly used additives in packaging are plasticizers, stabilizers, flame retardants, and fillers. Organic or inorganic additives are commonly employed to improve polymeric film properties like mechanical strength, stiffness, and gas barrier, addressing their inherent limitations [33,63]. The additive/matrix interfacial adhesion surely has an important influence in terms of the final mechanical properties of the composite film [64,65]. Incorporating fillers—mainly if nanostructured—can be a cost-effective way to improve gas barrier performances, to which factors like dispersion levels and nano-filler orientations contribute [66]. A key challenge generally is in effectively dispersing the additives within the matrix, mainly in common melt compounding with viscous polymers [67].

In this way, by employing additives and fillers, the life cycle may also be extended, leading to minor wastes. The additive concentration to increase the recycling value is also required to be relatively low, possibly below 10 wt%, according to some guidelines [68].

In the case of food packaging, a final consideration is related to the migration of additives into the products, for which specific national or international regulations exist in the case of potentially harmful additives [63].

### 1.4. Zeolites: Structure and Properties

Zeolites are microporous crystalline aluminosilicates that can also be found in nature and are structured as TO_4_ tetrahedra (T = Si, Al), with Si/Al ≥ 1. Their composition consists of a main framework, [Si_1-n_Al_n_O_2_], then an eventual extra framework of exchangeable, poorly bonded alkaline/alkaline earth cations such as Na^+^ (balancing the negative charge of the AlO_4_ tetrahedra), and a nH_2_O sorbed phase [69,70]. A zeolite’s framework type indicates the symmetric connectivity of its tetrahedrally coordinated atoms, without considering its structure, composition, or cell dimensions, as it happens with specific framework structures. Each framework type has a three-letter code based on its reference zeolite’s name. Zeolites can be naturally found in minerals, e.g., clinoptilolite, that belong to the Heulandite (HEU) family. The most important zeolites, however, are synthetic. Among those of interest, there are zeolites A, X, Y, and ZSM-5. Zeolite A is the reference for Linde Type A (LTA) zeolites, zeolites X and Y belong to the Faujasite (FAU) mineral family, and ZMS-5 (Zeolite Socony Mobil—five) is the reference material for the MFI family. So, zeolites are generally classified according to their structural nature (based on pore size) or acidic (based on the Si/Al ratio) nature [71,72,73,74,75]. For a given zeolite, the average pore measurement in Angstrom can precede the zeolite type name (e.g., zeolite 4A and zeolite 13X, respectively, are zeolite A with 4 Å pore size and zeolite X with 13 Å pore size), according to recommended nomenclature [71].

**Figure 2:**
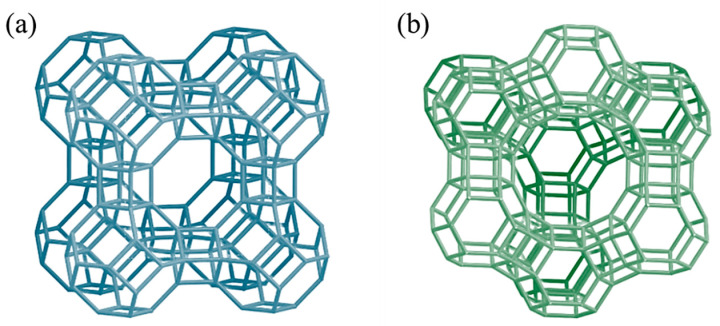
Frameworks of (**a**) zeolite A (LTA) and (**b**) zeolites X and Y (FAU) [86].

Zeolites are valid additives as they provide outstanding properties; these materials have unique and valuable possibilities of application thanks to the extensive inner surfaces and the resulting adsorption properties, combined with molecular sieving and cation exchange abilities [70]. The zeolite structure has a negative charge balanced, as mentioned, by alkaline/alkaline earth cations (mainly Na^+^ or K^+^), which can be exchanged by desired positive ions after immersing zeolites in properly ionized aqueous solutions. For example, it is possible to load zeolites with ions like Ag^+^, Zn^2+^, and Cu^2+^, which were proven to have antimicrobial (antibacterial, antifungal) activity [76,77]. Zeolites with low Si/Al ratios, which means zeolites with higher polarity and hydrophilicity, lead to high ion-exchange capacity; furthermore, in terms of selectivity, monovalent cations are preferred with low Si/Al ratios, whereas divalent cations are preferred with higher Si/Al ratios [78]. Regarding some of the most used synthetic zeolites, zeolite 4A has a Si/Al = 1, zeolite X has a Si/Al~1.24, and for zeolite Y, the Si/Al ratio is around 2.5–2.8; meanwhile, for ZSM-5, the Si/Al ratio is in a broad range with sensibly higher values (at least one order of magnitude bigger) [70,79,80]. In addition, different zeolite structures also have different porosity: the higher porosity of zeolites X and Y compared to zeolite A can be appreciated in Figure 2.

Furthermore, in terms of gas permeability—potentially with selective barrier properties—zeolites block gas molecules larger than their pores, permitting only smaller gases to pass through; adsorption effects on permeability at low temperatures have to be considered as well [81]. Zeolites can also be applied as ethylene scavengers due to their high surface on which they can selectively adsorb this gas. This is useful since the ethylene produced by climacteric fruits and vegetables (which are affected by post-harvest ripening) can reduce the shelf life of the fresh food itself [82,83]. 

Finally, regarding possible additive migration towards the packaged product, it is worth noting that the natural zeolite clinoptilolite is the only one to have a general recognition as completely safe and non-toxic among all zeolites, actually being widely and commonly used for applications that involve both animals and humans. Still, considering the very small amounts required for packaging films, other zeolites may be viable; surely, biological studies are required case by case to certify a possible application [84,85].

### 1.5. Objective of the Present Review

Extensive research has focused on the use of zeolites as additives in several polymeric media. In some reviews, the use of zeolites has been discussed in polymeric membranes for applications in water purification, catalysis, and gas separation; nevertheless, specific outcomes can also be helpful in understanding the behavior of zeolites in packaging films [87,88,89]. Furthermore, overviews on innovative (including active and nanostructured) additives for conventional and biodegradable polymeric packaging films were presented in other papers, although without providing specific insights on zeolites, cited among other additives [90,91,92,93]. Finally, other reviews focused exclusively on zeolite additives, particularly for packaging applications, but either they are outdated and do not include recent trends in biodegradable polymers or they discuss zeolites as active additives without a detailed study of mechanical, barrier, and thermal properties as well [94,95,96].

**Figure 3:**
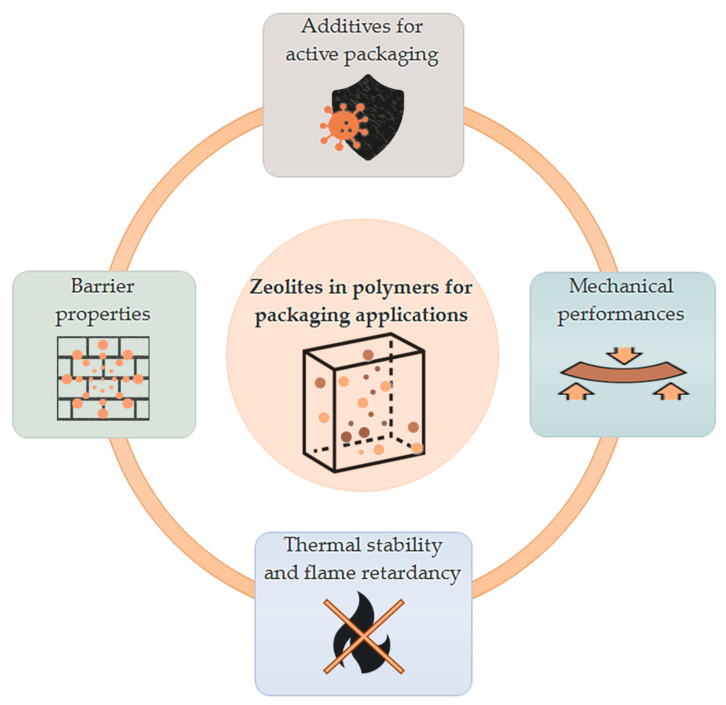
Scheme of the effects of zeolite additives on the properties of packaging polymers.

The following work focuses on recent trends in the use of both natural and synthetic zeolites as additives in polymers that actually are or that can be potentially applied in packaging, namely, PE, PP, and eco-friendly (bio-based/biodegradable) polymers, in order to valorize them in terms of performances and durability, both as flexible films and coatings. So, in addition to other literature reviews, the present work aims to give an overview of interesting and recently studied polymer/zeolite blends specifically for packaging film/coating applications, highlighting and determining both the positive and negative effects given by zeolite additives. The matrices considered in this review are mono-material; therefore, polymer blends are excluded. Also, only studies that employ zeolites as the main additives in terms of concentrations are considered. High interest is given to papers in which zeolites led to enhancements, as reported in Figure 3, regarding mechanical performance, thermal stability, and barrier to gases, additionally with scavenging properties and antimicrobial activity in the case of cation-exchanged zeolites. Where possible and relevant, the effects of zeolite additives on various neat film properties are reported in the present review as percentage differences (zeolite-blended vs. neat films), which are calculated from the values that were either explicitly indicated in each literature work or extrapolated from corresponding data graphs. 

It is worth noting that, according to its physicochemical structure, each polymer has a certain compatibility with given zeolites (whose polarity increases with the Si/Al ratio), and this compatibility affects the matrix–additive adhesion and so the final composite properties as well [97]. Improvements and new functionalities derived from these hybrid inorganic–organic composites, along with possible technological limits with respect to neat polymers, are highlighted.

## 2. Zeolite Additives in Polymers for Packaging

### 2.1. Zeolites in PE Films

High interest in the literature has been given to PE composites functionalized with zeolites. In Table 1, a summary of the different literature works is presented; the effects on neat PE film properties as percentage differences are reported as well.

In a less recent study that is worth mentioning, LDPE composites filled with micronized clinoptilolite natural zeolite were prepared by the melt extrusion method, with an optimal zeolite concentration of 4 wt%. Regarding mechanical properties, tensile strength increased, whereas elongation at break decreased. The most relevant effect was related to the gas permeability, with a noticeable enhancement in O_2_ and CO_2_ barriers. N_2_ permeability increased, but it can be considered harmless. The thermal stability of the films was similar, just slightly decreased [98].

To exploit its adsorbent capacity, mordenite natural zeolite with 4.6 wt% Cu was employed as filler for LDPE, working as an ethylene (C_2_H_4_) scavenger—a gas produced by fruits and vegetables that affects their shelf life when packaged. LDPE films with mordenite (up to 10 wt%) were obtained via extrusion. After 100 h of scavenging tests, the authors observed a reduction of 37% in the initial ethylene gas in the packaging headspace for the films with 10 wt% zeolite, due to the mordenite porous structure and the presence of copper that enhanced adsorption properties [99].

Chabazite-type natural zeolite exchanged with Ag-Cu-Zn was employed as a filler (1–15 wt%) for Bio-PE. The strategy involved an ion-exchange solution process for chabazite and then melt compounding extrusion with HDPE. Specifically, the elemental composition of zeolite revealed that the Ag^+^ concentration was about 1.4%, Cu^2+^ 0.3%, and Zn^2+^ 0.1%. The authors achieved antimicrobial properties against several bacteria (e.g., Staphylococcus and Escherichia, up to 100% inhibition) thanks to multi-ion zeolite, but also superior elastic modulus and hardness than unfilled Bio-PE. Nevertheless, the lower elongation at break was unsuitable for food packaging, and there were concerns from the authors about the potential toxicity related to Ag^+^ migration into food [100].

Furthermore, Ag-exchanged Ecuadorian natural zeolite was employed as an additive (1–3 wt%) for LDPE films, using the following procedure: cationic exchange (around 4% capacity), mixing with matrix, and heat pressing. Positive antimicrobial activity against *E. coli* bacteria was obtained due to Ag exchange, whereas an adverse effect on mechanical performances was shown in terms of reduced tensile strength and elongation at break compared to neat LDPE. Still, adding 2 wt% Ag–zeolite led to less negative impacts on mechanical properties, mainly if compared to adding natural non-exchanged zeolite [101].

Similarly, regarding synthetic zeolites, in a study, zeolite A was exchanged with Ag and chosen as an additive for PE films. The technique was cationic exchange for the zeolite and then wet-casting film after solubilization of PE with the zeolite in xylol. The authors achieved antimicrobial activity thanks to Ag^+^, and the best combinations were 5 wt% zeolites loaded with 5% and 10% silver. However, the authors underlined their concerns about the toxicity of this ion, like in the previously mentioned study; furthermore, a reduced crystallinity resulted. Additionally, the lowest antimicrobial activity occurred using hot casting, due to the high temperatures that may have affected the stability of Ag (causing its reduction), but also due to the non-homogeneous distribution in the film, with the additives not completely exposed on the PE surface. No effects of zeolites on any mechanical or barrier properties were analyzed [102]. 

A study concerning the flame retardancy of synthetic zeolite 4A in HDPE was carried out as well. The authors prepared polymeric systems via melt blending by combining two flame retardants—ammonium polyphosphate (APP) and tris-(2-hydroxyethyl)-isocyanurate—with the zeolite to evaluate its possible synergistic effect. The authors showed that the zeolite helped to further decrease the total smoke production, CO_2_ evolution, and degradation of the HDPE matrix; no significant deterioration in mechanical performance occurred with low zeolite content. Particularly, for the best formulation with the FRs and a 0.5 wt% zeolite, the limiting oxygen index (LOI) reached a maximum value of 26.3% from the original 18.5% of neat HDPE [103]. 

In the most recent work regarding this class of polymers, LDPE films were filled with edible natural zeolite, namely, Serbian micronized zeolite (5–10 wt%), by the extrusion molding process. The optimal concentration for zeolite was found to be 5 wt%, leading to an increase in the elastic modulus, ultimate strength, and O_2_ barrier. Elongation at break was similar; however, a slight reduction in the H_2_O barrier was observed. Lower elongation at breaks was obtained using 10 wt% concentration, even though it returned the best O_2_ barrier among tested samples [104].

**Table 1:** Studies on the effects of zeolite additives in PE films, in chronological order for LDPE and HDPE matrices. The optimal zeolite concentrations are reported.

Matrix	Zeolite Additive	% Zeolite	Effects (% Diff.)	Source
LDPE	Clinoptilolite	4 wt%	Tensile strength (+19%), barrier to O_2_ (+64%) and CO_2_ (+76%), elongation at break (−83%)	[98]
LDPE	Mordenite	10 wt%	Ethylene adsorption: q_max_ = 5.4 µL g^−1^	[99]
LDPE	Natural zeolite exch. with Ag^+^	2 wt%	Antimicrobial activity Tensile strength (−6%), elongation at break (−26%)	[101]
LDPE	Zeolite A exch. with Ag^+^	<5 wt%	Antimicrobial activity	[102]
LDPE	Natural zeolite	5 wt%	Elastic modulus (+34%), O_2_ barrier (+154%), antioxidant activity H_2_O barrier (−35%)	[104]
Bio-HDPE	Chabazite exch. with Ag-Cu-Zn	1–5 wt%	Antimicrobial activity, tensile modulus (+60% to +72%), hardness (+4% to +9%), elongation at break (−140% to −180%), tensile strength (−11% to −15%)	[100]
HDPE	Zeolite 4A	0.5 wt%	Flame retardancy, LOI (+35%, synergistic with other FRs)	[103]

### 2.2. Zeolites in PP Films

Insight is now given to the limited literature interest in the application of zeolites as additives in PP films. The calculated effects on neat PP films are reported in Table 2.

In a less recent but very interesting study on the investigated zeolite applications, zinc oxide nanoparticle (ZnO)-supported 13X zeolite additives were prepared by the ion exchange method and were used as multifunctional additives to be melt-blended with PP. The optimal content of ZnO-supported zeolites was 10 wt%. Ultraviolet (UV) resistance and antimicrobial activity, thanks to zeolites, were the main features of these functionalized PP composites [105].

In another study, PP was filled with either neat zeolite 4A or Ag-exchanged zeolite 4A (optimal 2.5 wt%). The procedure consisted of melt compounding in a twin-screw extruder and a consequent blowing process to obtain the films. While neat zeolite 4A was shown to give some protection against *S. aureus* but not against *E. coli*, Ag-zeolite 4A had the best antimicrobial activity against both bacteria. Due to the formation of voids, the elastic modulus was shown to decrease along with elongation at break and tensile strength, on which, however, the zeolite impact was lower. In general, neat zeolite/PP films were a bit more flexible than Ag–zeolite/PP films. Zeolite 2.5 wt% had a negligible effect on barriers to O_2_ and CO_2_ and on thermal stability. Also, the addition of a compatibilizer, maleic anhydride-grafted PP, had negligible enhancing effects on the barrier properties; the tensile strength and modulus increased, whereas elongation at break worsened [106]. 

In the previously discussed study related to Ag-exchanged natural Ecuadorian zeolites as an additive for LDPE films, the authors also prepared PP composites using a similar procedure. Along with the same efficient antimicrobial activity, the authors did not notice the worsening of mechanical performances that occurred with PE. Specifically, PP nanocomposites showed enhancements in tensile strength and elongation at break compared to neat PP, with the best-modified zeolite concentration set at 1 wt% [101]. 

More recently, PP-based films containing zeolite 4A and zinc oxide nanoparticles (ZnO NPs) were processed by the melt extrusion method. Particularly, ZnO clusters were incorporated into zeolite 4A through an ion exchange process (mixing zeolites and zinc acetate solution) and then calcinated. The results showed that the nanocomposites at 6 wt% additive concentration significantly increased the shelf life of the salmon that was enveloped in the PP-based film, also synergistically in the presence of green tea extract [107].

### 2.3. Zeolites in Biodegradable Plastics

In the current literature, there are several applications of zeolites in eco-friendly biodegradable polymeric matrices, as reported in Table 3, which includes the calculated percentage differences of the polymer properties affected by zeolite incorporation compared to neat films (as for the previously discussed PE-PP/zeolite films). Blends of different polymer matrices are not taken into account.

Chitosan has received high interest in the literature as a packaging polymer to be combined with zeolite additives. In a study, natural zeolite modified with Ag^+^ was used as an additive for chitosan to evaluate possible active packaging applications. The procedure required an ion exchange method for Ag^+^ into zeolite, followed by a solution casting method (2 wt% chitosan in 90% acetic acid solution + up to 2 wt% zeolite) onto a polystyrene Petri dish to obtain films. Antimicrobial activity was effectively achieved; furthermore, biocompatibility for chitosan and zeolite particles and the overall compatibility of chitosan–Ag–zeolite films were underlined by the authors [108]. 

A different filling for chitosan films, namely, the natural zeolite clinoptilolite, was studied by other authors. They started with film-forming solutions in water, composed of chitosan 1.5 to 2% *w*/*v*, lactic acid 1 wt%, and 3.5 wt% zeolite vs. chitosan; the solutions were then cast on glass plates. The obtained films were provided with high resistance to water solubility compared to neat chitosan films and shared similar opacity, but with lower elongation at break, tensile strength, and brightness. Particularly, 3.5 wt% zeolite in 1.75% *w*/*v* chitosan minimized and almost nullified the negative effects on mechanical properties, whereas 1.5% *w*/*v* chitosan with 3.5 wt% zeolite was the only to show an increase in the barrier to H_2_O [109]. 

Clinoptilolite as an additive in chitosan films was employed as well in another study, in this case, with the main function of an ethylene scavenger. A chitosan solution of 2% *m*/*v* was prepared in acetic acid 6% *v*/*v*; then, it was mixed with clinoptilolite, and the films were solvent cast with different methods. The final clinoptilolite content in the films that they studied was relatively high, 33 wt%. Water vapor permeability increased by adding zeolite compared to neat dense chitosan films. Furthermore, the authors studied ethylene scavenging capacity. The zeolite was regenerated either at 90 °C in the film or at 300 °C alone as a comparison, thus showing an ethylene adsorption effect for chitosan films that was slightly present for neat dense films but that further increased by adding zeolite [110].

In terms of coatings for active packaging, an interesting application for chitosan was suggested in a study, in which authors dispersed chitosan (up to 2.0%, *w*/*w*) in aqueous acetic acid; then, they cation-exchanged zeolite Y with Ag and added this modified zeolite to the suspension, which was later homogenized. Finally, an automatic applicator was used to obtain a dried coating for a packaging paper substrate. The wet coating formulation was varied for both the chitosan and zeolite. One aim of the research team was to evaluate the ethylene scavenging properties of the coatings, and they found good performance as active packaging when 10 wt% zeolite concentration [111].

Another interesting contribution in the field of biodegradable packaging films regarded the functionalization of fish gelatin using zeolite A modified with Zn^2+^. The steps followed by the authors were the ion exchange of zeolite (derived from glass waste and aluminum scrap) with a zinc acetate solution, then mixing modified zeolites (4–6 wt%) with gelatin and glycerol in water, and finally casting on a plastic plate. They observed an increase in thermal stability, antibacterial and antioxidant properties, and a better resistance to water solubility due to the very good gelatin–zeolite interaction [112].

Moving to other biodegradable polymers, PBS-based films filled with various zeolites (4A, Y, ZSM-5) modified with Ag^+^ were studied. After the ion exchange of zeolites with silver nitrate, two routes were followed to obtain the film: melt extrusion with dried PBS pellets and mixing in chloroform and solvent casting on a glass plate. The additive concentrations were 0.5–4 wt%. Via solvent casting, higher antibacterial activity (noticeable even at low Ag^+^–zeolite concentration) but lower mechanical properties were obtained compared to melt extrusion for all films, besides possible safety issues related to chloroform in solvent casting. Considering the extruded samples, the PBS films with ZSM-5 returned higher antibacterial efficiency than the ones with zeolites A and Y. Furthermore, the addition of zeolite Y or, mainly, the addition of ZSM-5 led to lower thermal stability of the films. Instead, adverse effects on the temperature of maximum degradation were negligible for samples with zeolite A. Finally, to keep an acceptable biodegradability rate of the composite films, a maximum of 0.5 wt% zeolite in melt blending was required [113].

HM pectin extracted from citrus and apples was investigated in a study as a polymer for food packaging films to be filled with zeolite Y. Exploiting its water solubility, pectin was dissolved in water, and polyglycerol was added to the solution as a plasticizer. Next, aqueous dispersions of zeolite Y, properly sonicated, were added to the film-forming solutions. The pectin (2 wt%) and polyglycerol (30 wt%) content remained constant, while the zeolite content in solution was varied from 0.05 wt% to 0.2 wt%—corresponding to 2.5 wt% and 10 wt% of total pectin. Films were obtained by casting and drying. The team found out that the 0.2 wt% zeolite Y in citrus pectin improved the tensile strength by 66%, thermal stability by 13%, and H_2_O barrier by 54%, highlighting the need for HM pectin to maximize interactions between polymer chains and zeolite Y. They used the films to pack strawberries, which effectively helped to maintain fruit color and appearance for longer time [114].

Another important packaging polymer is PLA. Some authors filled PLA with micro/nano-zeolites (4 wt%) activated by Cu. Melt mixing with hot-pressing onto a Teflon template with the desired thickness was used to obtain samples. In the case of micro-zeolites, the observed enhancement involved slightly higher O_2_ and H_2_O barrier properties and antimicrobial activity, even though no significant effects were observed on the mechanical properties. A stabilizer, polyethylene glycol (PEG), was instead required in the case of nano-zeolites to avoid agglomerations. Adding PEG did indeed allow for a strong increase in elastic modulus and tensile strength; however, the zeolite-compounded films became much more permeable to water vapor and oxygen than the neat films (unless using a specific PEG with a M_w_ of 1000 g/mol that kept the same barrier properties of neat films) [115]. 

To further enhance the proper dispersion of zeolites into PLA films (due to poor compatibility), other authors treated zeolite 3A to graft lactide onto it. They used HCl to remove Al^3+^ and ultrasonic treatment to reduce particle size; then, lactide was grafted on the zeolite by reacting with OH groups, with further polymerization of grafted PLA on the zeolite surface. Melt blending allowed the mixing of PLA with modified zeolite 3A (2 wt%) and epoxidized soybean oil (ESO) as a plasticizer (3 wt%) to later obtain film samples, which were compared to both neat PLA films and films with unmodified zeolite. A better dispersion was effectively achieved, leading to enhanced crystallinity and thermal stability, higher tensile strength and elongation at break, and improved barrier to both water vapor and oxygen. Particularly, the treatment was crucial to increasing gas barriers compared to the decrease with untreated filler [116].

In order to bypass technological limits related to starch-based films, zeolite ZSM-5 was incorporated as an additive in TPS in a less recent study that is worth mentioning. The TPS composition was 69% starch, 30% glycerol, and 1% stearic acid. Melt compounding was used to mix TPS with zeolites (2–10 wt%) and then thermo-pressing to obtain films. An increase in water vapor barrier, elastic modulus, and tensile strength was observed, with a slight decrease in elongation at break. The best concentration was 2 wt% in terms of overall mechanical properties and water vapor barrier [117].

More recently, for the same kind of matrix, zeolite A was embedded in starch-based films. The authors used TPS consisting of 70% corn starch and 30% glycerol. Then, after twin-screw extruder processing, the composite films with zeolite A (4 wt%) were obtained. Zeolite A effectively worked as a reinforcing filler and was also shown to interfere with or destroy H-bonding interactions in TPS. In particular, compared to the neat polymer, the composite had a good increase in tensile strength, elastic modulus, tenacity, and elongation at break. However, there was no impact on water sensitivity [118].

## 3. Conclusions

The implementation of zeolites as innovative additives for flexible packaging polymers, particularly for conventional polyolefins (PE, PP) and for some biodegradable polymers, has been detailed. The preparation and characterization of zeolite/polymer blends have been reported, determining the effect of zeolite additives on the neat film properties.

Regarding PE films, studies with zeolite additives revealed promising antimicrobial, gas scavenging, and gas barrier properties for food packaging applications. Ag-exchanged zeolite in LDPE effectively inhibited bacteria growth, though it impacted some mechanical properties. Other zeolites, like clinoptilolite and mordenite, improved gas permeability and tensile strength but reduced flexibility. Mordenite with Cu served as an ethylene scavenger, extending produce shelf life, while multi-ion chabazite provided broad antimicrobial effects, but with concerns about ion migration and reduced elasticity. A synergistic effect with flame retardants was highlighted as well. 

For PP films, ZnO-supported 13X zeolite also showed promising UV and microbial resistance, while Ag-exchanged zeolite additives enhanced antimicrobial activity without the mechanical drawbacks that were observed in PE by the same authors, improving tensile strength and elongation at break instead. Other authors observed that neat or Ag-exchanged zeolite 4A slightly reduced mechanical properties. Additionally, PP films with zeolite 4A and embedded ZnO NPs significantly contributed to extending food shelf life.

Regarding various green polymers, ZSM-5, zeolite A, and clinoptilolite additives improved the H_2_O barrier, mechanical strength, and antimicrobial properties of starch, PLA, chitosan, and pectin films. Furthermore, ethylene scavenging, higher gas barriers, and thermal stability were achieved. Films modified with Ag or Zn ions, like those reported for PBS and gelatin matrices, also showed notable antibacterial effects.

Therefore, zeolites surely represent a potentially valid alternative to conventional fillers in packaging films, with the most promising application to be possibly found to enhance green packaging properties. However, some challenges are still present to reduce or limit some drawbacks for composites that may occur according to the preparation and to the employed procedure, matrix, and zeolite concentration, mainly due to phenomena like matrix/additive incompatibility, uneven mixing, and nano-filler agglomeration.

More research is needed on PP/zeolite blends, as the literature lacks sufficient papers regarding what is the second most used plastic worldwide for packaging application, and also, none of these papers analyzed the effects of zeolite additives on the water vapor barrier properties of PP. Analogously, PE/zeolite films should be studied more in-depth to evaluate the effect on water vapor permeability, as this property is crucial for many packaging applications and only one study evaluated the outcome. Recyclability tests would be interesting for these polyolefin-based systems as well. 

Other suggestions regard the research on biodegradable polymers. Since the mechanical properties of these polymers tend to be lower than those of polyolefins, more tensile/flexural tests should be carried out, mainly on chitosan, PBS, and gelatin films with zeolite additives, as the effect would be of high interest for the final packaging applications. Additionally, biodegradability tests should be conducted on most of these hybrid blends to evaluate if the sustainability of these composites can be kept overall.

Finally, many studies have been concerned with the antimicrobial activity of cation-exchanged zeolites in polymeric films. A general research prospect may focus on another side of active packaging that zeolites can offer, namely, gas adsorption. Just a few papers have investigated this property. The maximum temperatures at which these polymer films can be processed is a limiting parameter for the complete regeneration and consequent adsorption properties of zeolites. Still, depending on the polymeric matrix, this activity may be evaluated and optimized, as it could help in many packaging applications.

## Figures and Tables

**Table 2:** Studies on the effects of zeolite additives in PP films, in chronological order. The optimal zeolite concentrations are reported.

Matrix	Zeolite Additive	% Zeolite	Effects (% Diff.)	Source
PP	Zeolite 13X ZnO supported	10 wt%	UV resistance, antimicrobial activity	[105]
PP	Zeolite A, neat & exch. with Ag^+^	2.5 wt%	Antimicrobial activity, crystallinity Elastic modulus (up to −24%), elongation at break (up to −19%)	[106]
PP	Natural zeolite exch. with Ag^+^	1 wt%	Antimicrobial activity, tensile strength (+60%), elongation at break (+114%), thermal stability: T_degr_ from 343 to 408 °C	[101]
PP	Zeolite 4A w/ ZnO NPs clusters	6 wt%	Antimicrobial activity (synergistic with green tea extract)	[107]

**Table 3:** Studies on the effects of zeolite additives in biodegradable polymer films, in chronological order for each considered matrix. The optimal zeolite concentrations are reported.

Matrix	Zeolite Additive	% Zeolite	Effects (% Diff.)	Source
Chitosan	Natural zeolite exch. with Ag^+^	<2 wt%	Antimicrobial activity, biocompatibility	[108]
Chitosan (1.75% *w*/*v*)	Clinoptilolite	3.5 wt%	Resistance to water solubility (+42%) Tensile strength (up to −8%)	[109]
Chitosan	Clinoptilolite	33 wt%	C_2_H_4_ adsorption (+57%) H_2_O barrier (−118%)	[110]
Chitosan	Zeolite Y exch. with Ag^+^	10–20 wt%	C_2_H_4_ adsorption: 125 µmol/g (coating on poorly adsorbent paper)	[111]
Gelatin	Zeolite A exch. with Zn^2+^	4–6 wt%	Antioxidant (+8%) activity, antibacterial activity, thermal stability, resistance to water solubility (+65% to 123%),	[112]
PBS	Zeolites (4A, Y, ZSM-5) exch. with Ag^+^	0.5 wt%	Antibacterial activity (best for ZSM-5) Temperature of max degradation (−6.5% for ZMS-5, −3.5% for Y, no effect for A), biodegradability (−143% after 5 months)	[113]
Pectin	Zeolite Y	0.2 wt%	Tensile strength (+66%), H_2_O barrier (+54%), thermal stability (+13%)	[114]
PLA	Micro-zeolites exch. with Cu	4 wt%	Barrier to O_2_ (+11%) and H_2_O (+13%), elastic modulus (+11%), antimicrobial activity	[115]
PLA	Lactide-grafted zeolite 3A	2 wt%	Crystallinity (+37%), elongation at break (+133%), tensile strength (+22%), thermal stability: T_init.degr._ from 302 to 330 °C, barrier to O_2_ (+3%) and H_2_O (+20%).	[116]
TPS	Zeolite ZSM-5	4 wt%	Elastic modulus (+115%), ultimate tensile strength (+61%), H_2_O barrier (+6%) Elongation at break (−17%)	[117]
TPS	Zeolite A	4 wt%	Tensile strength (+90%), Young modulus (+47%), elongation at break (+63%), tenacity (+140%)	[118]
